# Non-invasive identification of high-risk plaques in non-culprit lesions of acute coronary syndrome

**DOI:** 10.1007/s00380-026-02663-6

**Published:** 2026-02-16

**Authors:** Kazuyoshi Kakehi, Masafumi Ueno, Masaru Kayama, Kei Hamanaka, Haruka Minami, Nobuhiro Yamada, Kyohei Onishi, Keishiro Sugimoto, Yohei Funauchi, Takayuki Kawamura, Kosuke Fujita, Koichiro Matsumura, Gaku Nakazawa

**Affiliations:** Division of Cardiology, Department of Internal Medicine, Kindai University Faculty of Medicine, Ohno-Higashi, Osakasayama, Osaka 589-8511 Japan

**Keywords:** Acute coronary syndrome, High-risk plaque, Coronary computed tomographic angiography, Maximum lipid core burden index

## Abstract

Identifying high-risk plaques (HRPs) using non-invasive imaging modalities is clinically important. The diagnostic value of combining coronary computed tomography angiography (CCTA)–derived measurements with fractional flow reserve derived from computed tomography (FFR_CT_) for detecting HRPs in non-culprit lesions of acute coronary syndrome (ACS) remains to be clarified. This study aimed to assess whether ΔFFR_CT_ and CCTA-derived plaque features can accurately identify HRPs detected by near-infrared spectroscopy and intravascular ultrasound (NIRS-IVUS) in non-culprit lesions of ACS. We prospectively evaluated 105 non-culprit coronary lesions using CCTA, FFR_CT_, and NIRS-IVUS in 32 patients with ACS. ΔFFR_CT_ was calculated as the difference in FFR_CT_ across the stenosis. Receiver operating characteristic (ROC) analysis determined the optimal cutoff values of ΔFFR_CT_ and CCTA-derived plaque features for predicting a maximum 4-mm lipid core burden index (maxLCBI_4mm_) ≥ 400. Both ΔFFR_CT_ values and CCTA-derived plaque features were associated with a maxLCBI_4mm_ ≥ 400 (both *P* < 0.05). The optimal cutoff values of ΔFFR_CT_ and plaque density for predicting a maxLCBI_4mm_ ≥ 400 were 0.06 and 29 Hounsfield units, respectively. The combination of ΔFFR_CT_ ≥ 0.06 and low plaque density predicted a maxLCBI_4mm_ ≥ 400 with 89.1% sensitivity and 84.8% specificity (area under the curve = 0.90; *P* < 0.0001). In multivariate analysis, plaque density ≤ 29 HU and ΔFFR_CT_ ≥ 0.06 independently predicted a maxLCBI_4mm_≥ 400. The combination of ΔFFR_CT_ ≧0.06 and plaque density on CCTA predicted a maxLCBI_4mm_ ≧400 with 89.1% sensitivity and 84.8% specificity (area under the curve, 0.90; *P* < 0.0001). In multivariate analysis, plaque density ≦ 29 (odds ratio 7.72, 95% confidence interval 2.10–28.44, *P* = 0.002) and ΔFFR_CT_ ≧0.06 (4.55, 1.21–17.04, *P* = 0.02) were independent predictors of a maxLCBI_4mm_ ≧400. HRPs with a maxLCBI_4mm_ ≧400 can be diagnosed with high accuracy by confirming a plaque density ≦ 29 in lesions with ΔFFR_CT_ ≧0.06.

**Supplementary Information:**

The online version contains supplementary material available at https://doi.org/10.1007/s00380-026-02663-6.

## Introduction

Coronary artery disease remains a leading cause of mortality worldwide. Even with contemporary secondary prevention, patients with prior myocardial infarction remain at high risk for future cardiovascular events [1]. Autopsy studies have shown that many coronary events are triggered by plaque rupture, with thin-cap fibroatheroma recognized as its pathological precursor [2, 3]. Various intravascular imaging modalities have been developed to detect such precursor lesions. Near-infrared spectroscopy combined with intravascular ultrasound (NIRS-IVUS) enables quantitative assessment of lipid-rich plaques, which are characteristic of acute coronary syndrome (ACS) [4]. The lipid core burden index (LCBI), automatically derived from chemograms, quantifies lipid content within a vessel. Among these parameters, the maximum 4-mm lipid core burden index (maxLCBI_4mm_) has been validated as a robust marker of lipid-rich plaques [5], and a maxLCBI_4mm_ ≥ 400 corresponds to plaques prone to rupture and cause ACS [6]. Coronary computed tomographic angiography (CCTA) allows noninvasive evaluation of coronary plaques. In a prospective study with a 27-month follow-up, patients with plaques showing either low attenuation (< 30 Hounsfield units [HU]) or positive remodeling (remodeling index > 1.1) had a significantly higher incidence of ACS, particularly when both features coexisted [7]. Conversely, ACS was rare in patients without these features, suggesting that the comprehensive assessment of plaque vulnerability factors on CCTA may aid in risk stratification. Recent studies also support the use of CCTA for aggressive secondary prevention strategies [8].

Fractional flow reserve derived from computed tomography (FFR_CT_) is a novel technique that non-invasively estimates the hemodynamic significance of coronary stenosis based on computational fluid dynamics modeling of CCTA data. Previous studies comparing FFR_CT_ with invasive FFR have demonstrated that adding FFR_CT_ improves diagnostic specificity and accuracy [9]. Previous studies have reported that the incidence of future cardiovascular events is twice as high in non-culprit lesions with lipid-rich plaques as in culprit lesions [10], and even if the lesion does not restrict blood flow, the presence of vulnerable plaques increases the risk of future events [11]. Therefore, non-invasive identification of high-risk plaques (HRPs) in non-culprit lesions is clinically important for preventing recurrent events.

This study aimed to determine whether combining ΔFFR_CT_ with CCTA-derived plaque features could accurately identify lipid-rich plaques (maxLCBI_4mm_ ≥ 400) detected by NIRS-IVUS in non-culprit lesions of ACS.

## Methods

### Study population and design

In this prospective observational study, we compared maxLCBI_4mm_ and CCTA/FFR_CT_ findings in non-culprit lesions of ACS. Patients were recruited from the Division of Cardiology at Kindai University Hospital and were enrolled if they met all of the following criteria: (1) admission with ACS, including ST-segment elevation myocardial infarction, non-ST-segment elevation myocardial infarction, or unstable angina; (2) presence of intermediate stenosis in non-culprit lesions on coronary angiography or CCTA; (3) NIRS-IVUS evaluation for intermediate stenosis after percutaneous coronary intervention (PCI); and (4) CCTA and FFR_CT_ performed within 14 days of PCI.

Exclusion criteria were as follows: (1) chronic kidney disease with an estimated glomerular filtration rate < 30 mL/min/1.73 m²; (2) hemodynamic instability; (3) severe valvular disease; (4) left main trunk lesions; (5) lesions unsuitable for NIRS-IVUS assessment due to tortuosity, severe stenosis, or heavy calcification; and (6) poor image quality preventing adequate CCTA or NIRS-IVUS analysis.

Each coronary artery was divided into 30-mm segments beginning at the ostium to evaluate lesion vulnerability. A non-culprit lesion was defined as intermediate stenosis without a history of PCI and located in a different 30-mm segment from the culprit lesion. Intermediate stenosis was defined as > 25% stenosis on quantitative coronary angiography and 25–75% diameter stenosis on CCTA. Coronary angiography was performed via radial or femoral access using a 6 Fr catheter. The left anterior descending, left circumflex, and right coronary arteries were evaluated with NIRS-IVUS after treatment of the culprit lesion.

This study was approved by the Ethics Committee of Kindai University Hospital (approval No. R03-195) and conducted in accordance with the Declaration of Helsinki. Written informed consent was obtained from all participants.

### NIRS imaging

**Fig. 1:**
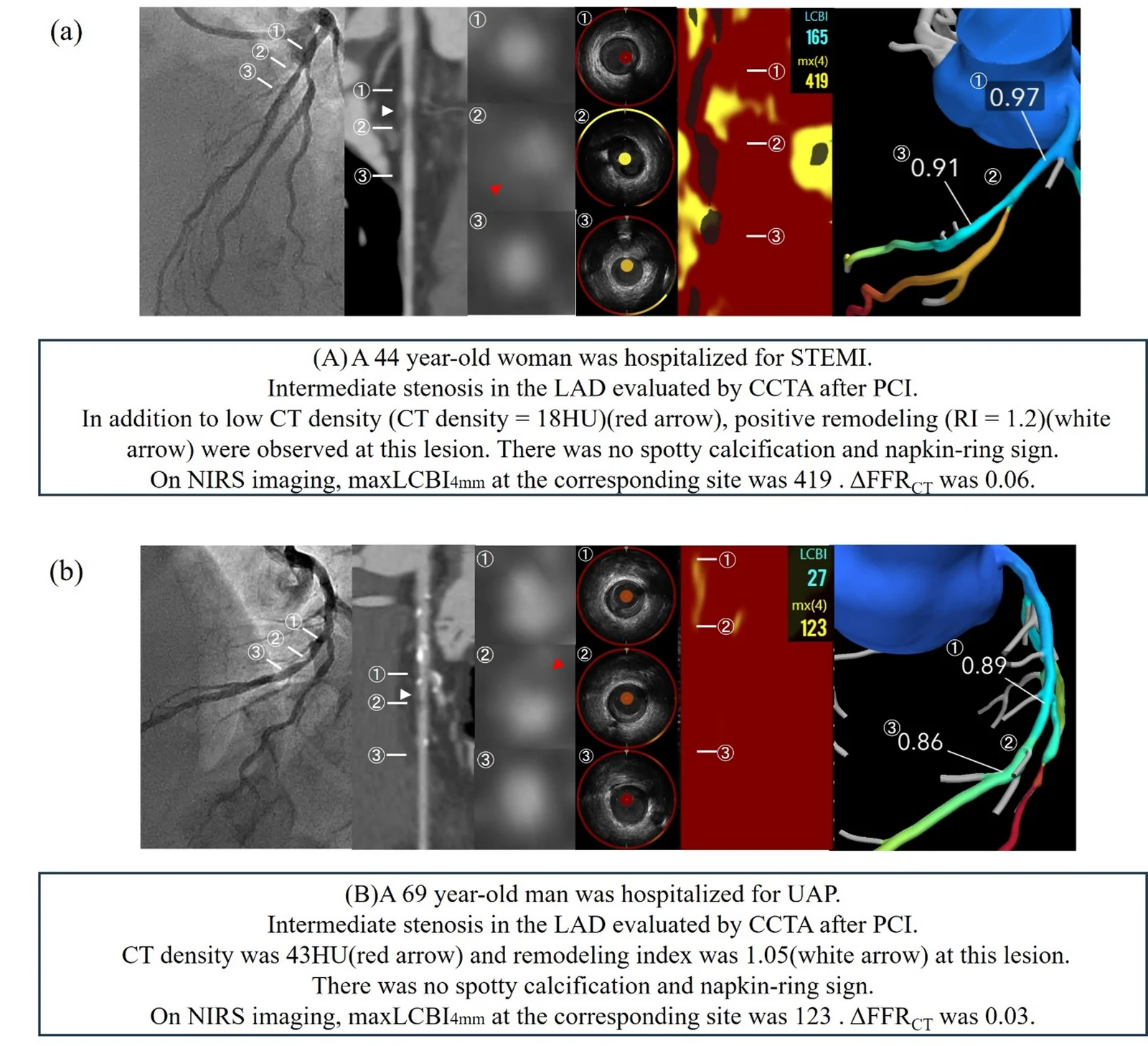
Representative cases. CCTA: coronary computed tomography, CT: computed tomography, FFR_CT_: fractional flow reserve derived from computed tomography, HU: Hounsfield units, LAD: left anterior descending, maxLCBI_4mm_: maximum 4-mm lipid-core burden index, NIRS: near-infrared spectroscopy, PCI: percutaneous coronary intervention, RI: remodeling index, STEMI: ST-elevation myocardial infarction, UAP: unstable angina pectoris

The NIRS system (Dual pro™, Infraredx, Bedford, MA, USA) consisted of a 3.2 Fr rapid-exchange catheter, a pullback and rotation device, and a console. Image acquisition was performed automatically with a pullback rate of 1.0 mm/s and rotation speed of 1,800 rpm. Areas with lipid-core characteristics were displayed in yellow on the chemogram. The Makoto^®^ system (Infraredx) was used to analyze the chemogram data [12]. Each coronary artery was divided into 30-mm segments from the ostium, and the maxLCBI_4mm_ within each segment was calculated. High-risk plaques (HRPs) were defined as maxLCBI_4mm_ ≥ 400 [13]. Two representative cases are shown in Fig. 1.

### CCTA imaging

Cardiac CT was performed using an electrocardiogram-gated 512-slice scanner (GE Healthcare, Chicago, IL, USA). Standard enhancement and imaging protocols were applied. Sublingual nitrates were administered before scanning, and β-blockers were used as needed to maintain a heart rate < 60 bpm. A 10-mL test bolus of iopamidol (Iopamiron 370; Bracco Diagnostics, Milan, Italy) was injected to determine scan timing, followed by a main bolus of 0.8 mL/kg. Scanning was triggered at maximal aortic enhancement. Acquisition parameters included 120 kV, 500–700 mA, helical pitch 0.16, gantry rotation 350 ms, and slice thickness 0.625 mm. Images were reconstructed at 75% of the RR interval.

Plaques were considered vulnerable if they exhibited: (1) low attenuation (< 30 HU), (2) positive remodeling (index ≥ 1.1), (3) spotty calcification (< 3 mm on curved multiplanar reconstruction), or (4) a napkin-ring sign [14]. The SYNAPSE VINCENT^®^ software (Fujifilm Medical, Tokyo, Japan) was used to measure plaque density, remodeling index, and calcification [15]. Plaque density (HU) was determined by averaging several regions of interest (0.5–1.0 mm² each). Remodeling index was defined as the vessel diameter at the plaque site divided by that at a proximal reference site; positive remodeling was an index ≥ 1.1.

### FFR_CT_

FFR_CT_ was computed from CCTA data using HeartFlow Inc. (Redwood City, CA, USA) with computational fluid dynamics–based modeling. Coronary blood flow and pressure were simulated under conditions of maximal hyperemia [16].

Delta (Δ) FFR_CT_ represented the pressure difference across a stenosis and was defined as the proximal FFR_CT_ minus the distal FFR_CT_ value (i.e., ΔFFR_CT_=proximal FFR_CT_−distal FFR_CT_).

The proximal and distal FFR_CT_ values used to calculate ΔFFR_CT_ were defined relative to the lesion boundaries identified on CCTA, rather than at a fixed distance.

### Statistical analysis

Continuous variables were tested for normality using the Kolmogorov–Smirnov test. Data are expressed as mean ± standard deviation for normally distributed variables or as median (interquartile range) otherwise. Between-group differences were assessed using Student’s t-test or the Mann–Whitney U test. Categorical variables are presented as counts and percentages and compared using the chi-square or Fisher’s exact test. Receiver operating characteristic (ROC) curve analysis determined optimal cutoff values of CCTA-derived plaque features and ΔFFR_CT_ for predicting maxLCBI_4mm_ ≥ 400. Multivariate logistic regression identified independent predictors of maxLCBI_4mm_ ≥ 400. Variables with *p* < 0.05 in univariate analysis or considered clinically relevant were included in the model. All *p* values < 0.05 were considered statistically significant. Statistical analyses were performed with JMP Pro (SAS Institute Inc., Cary, NC, USA).

## Results

### Study population

We enrolled 117 consecutive patients with intermediate stenosis in non-culprit lesions, as identified by coronary angiography or CCTA, who were admitted with ACS to Kindai University Hospital between December 2021 and January 2024. Of these, 85 patients were excluded for the following reasons: six patients had lesions that could not be assessed by NIRS-IVUS; 63 patients underwent coronary angiography but not CCTA; one patient had an interval of more than 14 days between CCTA and percutaneous coronary intervention; and 15 patients did not undergo FFR_CT_. Finally, 32 patients were included in the study (Fig. 1). Table 1 shows the patients’ characteristics. The mean age was 68 ± 11.5 years, 84.4% were men, and 53.1% had unstable angina. The prevalence of cardiovascular risk factors was high (hypertension, 78.1%; dyslipidemia, 71.9%; and type 2 diabetes mellitus, 40.6%). The mean low-density lipoprotein cholesterol concentration at admission was 117.6 ± 33.6 mg/dL.

**Table 1:** Patient characteristics

Basic characteristics	(*n* = 32 patients)
Age	68.0 ± 11.5
Male gender, n (%)	27 (84.4%)
Body mass index (kg/m^2^)	23.1 (21.3–24.8)
Hypertension, n (%)	25 (78.1%)
Diabetes mellitus, n (%)	13 (40.6%)
Dyslipidemia, n (%)	23 (71.9%)
Smoking (past, current), n (%)	22 (68.8%)
Clinical presentation	
STEMI, n (%)	12 (37.5%)
NSTEMI, n (%)	3 (9.4%)
UAP, n (%)	17 (53.1%)
Medication at admission	
β-blocker, n (%)	7 (21.9%)
ACE-I/ARB, n (%)	11 (34.4%)
Statin, n (%)	4 (12.5%)
Ezetimibe, n (%)	0 (0%)
Laboratory data	
eGFR(ml/min/1.73m^2^)	70.8 ± 19.3
HbA1c, (%, IQR)	6.1 (6.0-6.8)
Triglyceride, (mg/dl, IQR)	105 (64.5–118)
LDL-cholesterol (mg/dl)	117.6 ± 33.6
HDL-cholesterol (mg/dl)	48.7 ± 10.1
Non HDL-cholesterol (mg/dl)	138.8 ± 35.0

### CCTA imaging and NIRS-IVUS features of the analyzed lesions

Table 2 summarizes the angiographic characteristics of the analyzed lesions. A total of 43% of the analyzed lesions were located in the left anterior descending artery, and approximately 50% were proximal lesions.

**Table 2:** CCTA, NIRS measurements and functional indexes

Location of lesions	(*n* = 105 lesions)
LAD, n (%)	45 (42.9%)
LCX, n (%)	27 (25.7%)
RCA, n (%)	33 (31.4%)
Proximal lesion, n (%)	52 (49.5%)
Mid lesion, n (%)	38 (36.2%)
Distal lesion, n (%)	13 (12.4%)
Far distal lesion, n (%)	2 (1.9%)
CCTA findings at maxLCBI_4mm_ lumen	
Duration of days between CT and PCI, (days, IQR)	4.0 (0-8.8)
Vessel area, (mm^2^, IQR)	13.7 (9.9–16.7)
Lumen area, (mm^2^, IQR)	4.9 (3.3–6.6)
Plaque area, (mm^2^, IQR)	8.3 (5.9–10.8)
Plaque density, (HU, IQR)	41.0 (26.0–60.0)
% diameter stenosis, (%, IQR)	41.0 (30.0–51.0)
Remodeling index, (IQR)	1.0 (0.95–1.1)
Spotty calcification, n (%)	28 (26.7%)
Napkin-ring sign, n (%)	9 (8.6%)
NIRS-IVUS findings	
Plaque burden at the site of maxLCBI_4mm_ (%, IQR)	61 (47.5–68)
Minimal lumen area at the site of maxLCBI_4mm_ (mm^2^, IQR)	4.8 (3.6–6.7)
Plaque area with minimal lumen area at the site of maxLCBI_4mm_ (mm^2^, IQR)	7.6 (5.6–11.0)
MaxLCBI_4mm_, (IQR)	350 (220.5-483.5)
MaxLCBI_4mm_ ≧ 400	46 (43.8%)
Functional indexes at the site of maxLCBI_4mm_	
ΔFFR_CT_ (IQR)	0.05 (0.03–0.12)

**Fig. 2:**
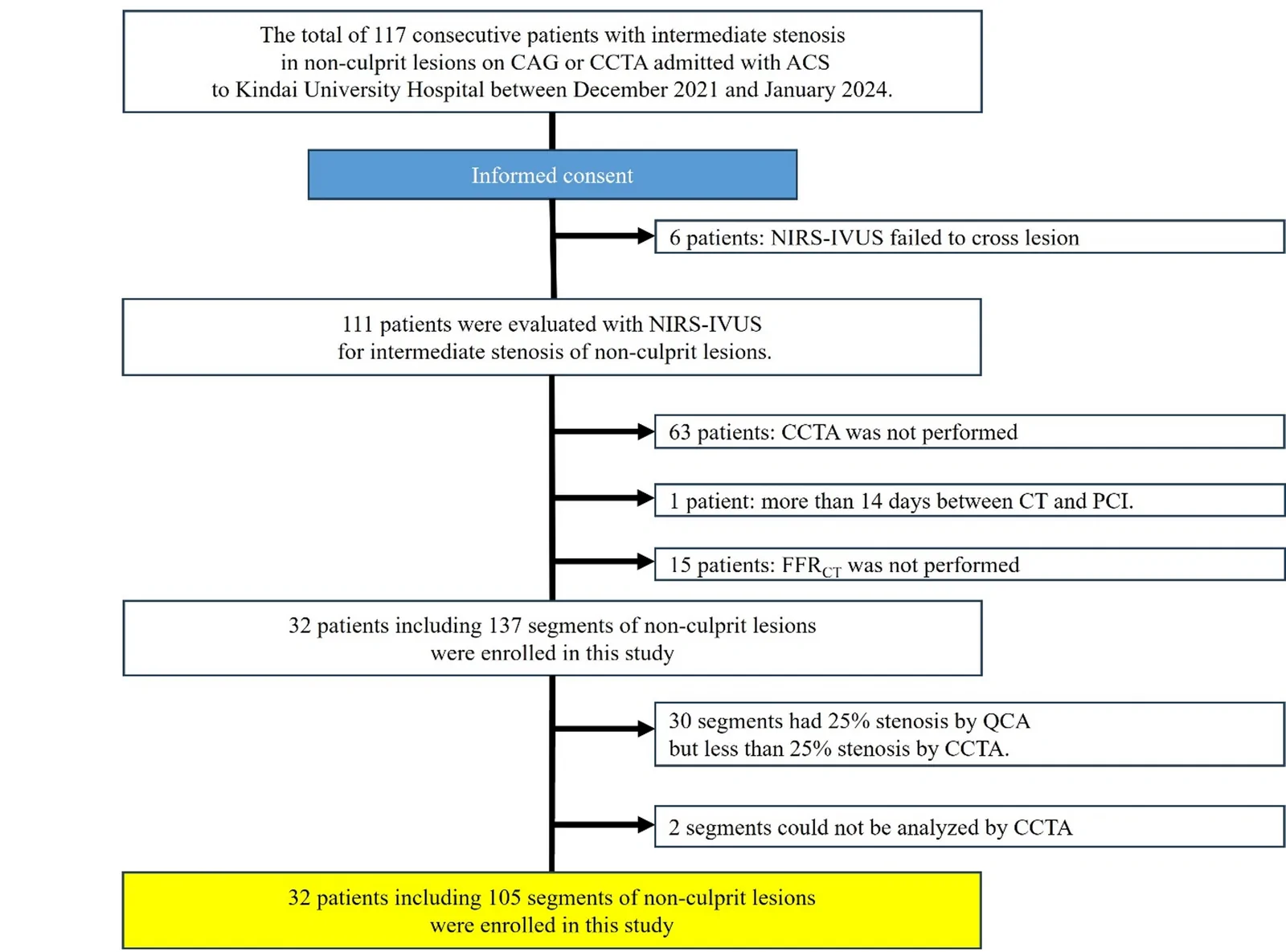
Study flow diagram. Flow diagram of patient selection and study design. A total of 32 consecutive patients with ACS underwent both CCTA and FFR_CT_ in addition to NIRS-IVUS evaluation. ACS: acute coronary syndrome, CAG: coronary angiography, CCTA: Coronary computed tomography, FFR_CT_: fractional flow reserve derived from computed tomography, NIRS-IVUS: near-infrared spectroscopy and intravascular ultrasound, PCI: percutaneous coronary intervention, QCA: quantitative coronary angiography

In the CCTA analysis, the median CT attenuation value of plaques within the 30-mm segment was 41 (26–60) HU, and the median remodeling index was 1.0 (0.95–1.1). Spotty calcification and the napkin-ring sign were observed in 26.7% and 8.6% of lesions, respectively. NIRS measurements and ΔFFR_CT_ values for the corresponding lesions are shown in Table 2. The median maxLCBI_4mm_ was 350 (221–484), and the prevalence of maxLCBI_4mm_ ≥ 400 was 43.8%. The median ΔFFR_CT_ was 0.05 (0.03–0.12) (Fig. 2).

The characteristics of CCTA, NIRS measurements, and the functional indices of the analyzed coronary lesions with and without maxLCBI_4mm_ ≥ 400 are presented in Supplemental Table 1.

### Relationship between maxLCBI_4mm_ and CCTA-derived plaque features

**Fig. 3:**
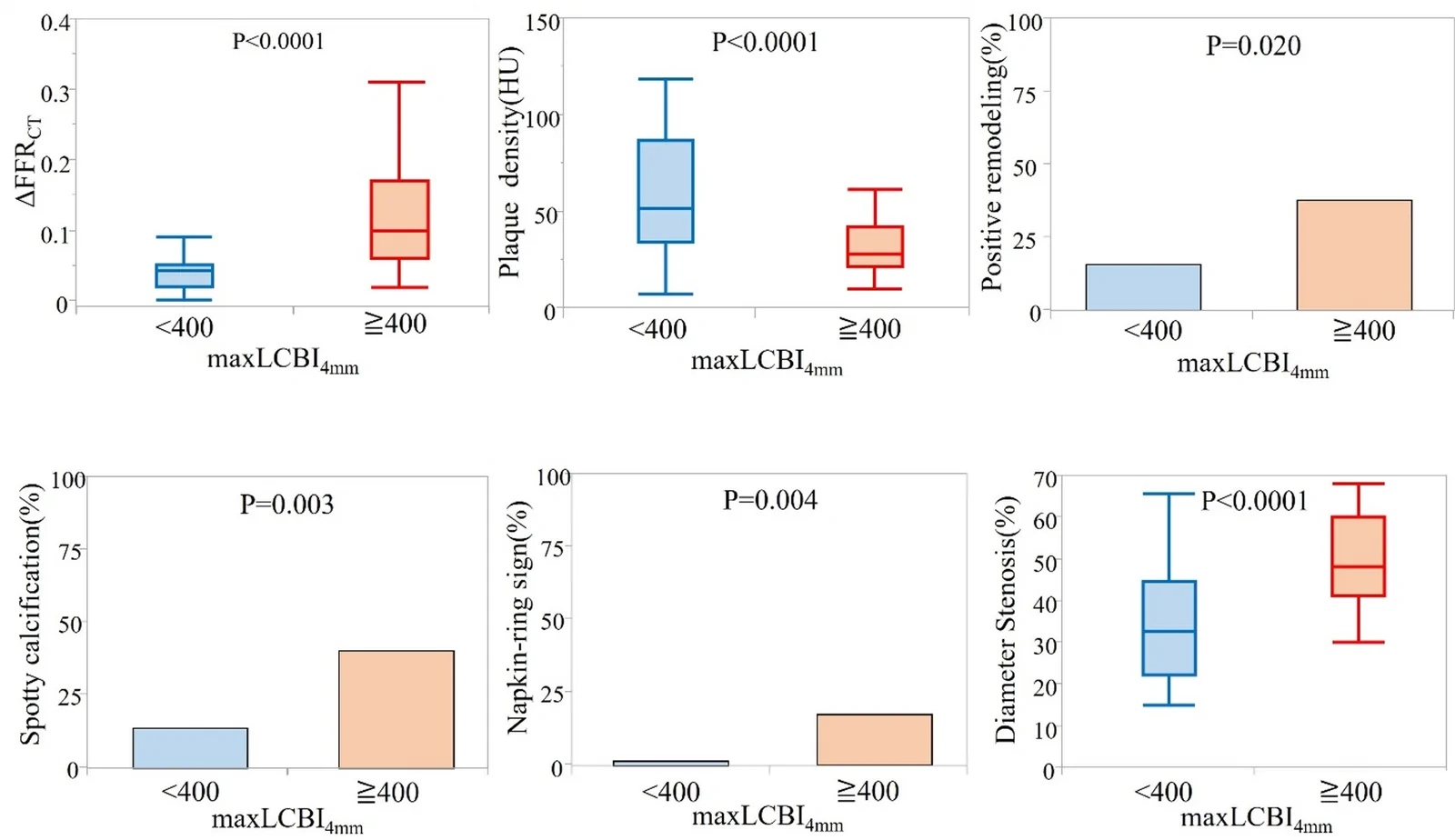
Comparison of CCTA and ΔFFR_CT_ measurements between analyzed coronary lesions with and without maxLCBI_4mm_ ≧400. Lesions with maxLCBI_4mm_ ≥ 400 showed significantly higher ΔFFR_CT_ values and lower plaque density, along with a higher frequency of positive remodeling, spotty calcification, and napkin-ring sign, as well as greater percent diameter stenosis on CCTA. CCTA: coronary computed tomography, FFR_CT_: fractional flow reserve derived from computed tomography, HU: Hounsfield units, maxLCBI_4mm_: maximum 4-mm lipid-core burden index

**Fig. 4:**
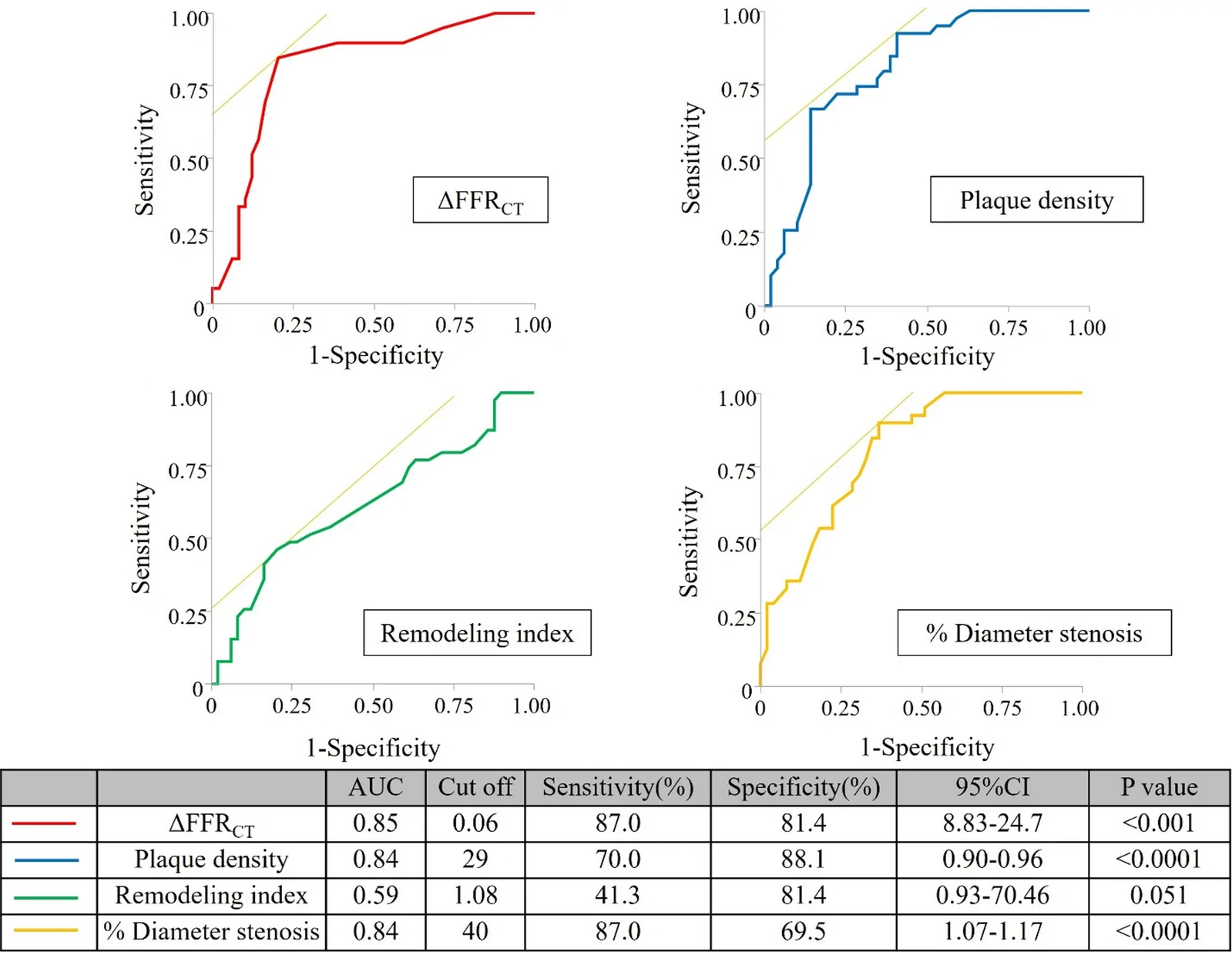
ROC curve analysis for predicting maxLCBI_4mm_ ≥400. ROC curve analysis was performed to evaluate ΔFFR_CT_, plaque density, remodeling index, and percent diameter stenosis for predicting maxLCBI_4mm_ ≥ 400. AUC: area under the curve, CI: confidence interval, FFR_CT_: fractional flow reserve derived from computed tomography, MaxLCBI_4mm_: maximum 4-mm lipid-core burden index, ROC: receiver operating characteristic

Figure 3 shows the relationships between maxLCBI_4mm_ and CCTA-derived plaque features. Lesions with maxLCBI_4mm_ ≥ 400 were more likely to have a higher ΔFFR_CT_ (0.10 [0.06–0.18] vs. 0.04 [0.02–0.05], *p* < 0.0001) and lower plaque density (28 [20–33] HU vs. 54 [41–76] HU, *p* < 0.0001), along with a higher frequency of positive remodeling (37.0% vs. 17.0%, *p* = 0.02), spotty calcification (41.3% vs. 15.3%, *p* = 0.003), and napkin-ring sign (17.0% vs. 1.7%, *p* = 0.004), and greater percent diameter stenosis on CCTA (50 [42–60] vs. 31 [25–45], *p* < 0.001). Figure 4 shows the ROC curves for predicting maxLCBI_4mm_ ≥ 400. The ROC analysis demonstrated that ΔFFR_CT_ ≥ 0.06 (area under the curve [AUC] = 0.85, sensitivity 87.0%, specificity 81.4%), plaque density ≤ 29 HU (AUC = 0.84, sensitivity 70.7%, specificity 88.1%), remodeling index ≥ 1.08 (AUC = 0.59, sensitivity 41.3%, specificity 81.4%), and percent diameter stenosis ≥ 40% (AUC = 0.84, sensitivity 87.0%, specificity 69.5%) were optimal cutoff values associated with maxLCBI_4mm_ ≥ 400.

**Fig. 5:**
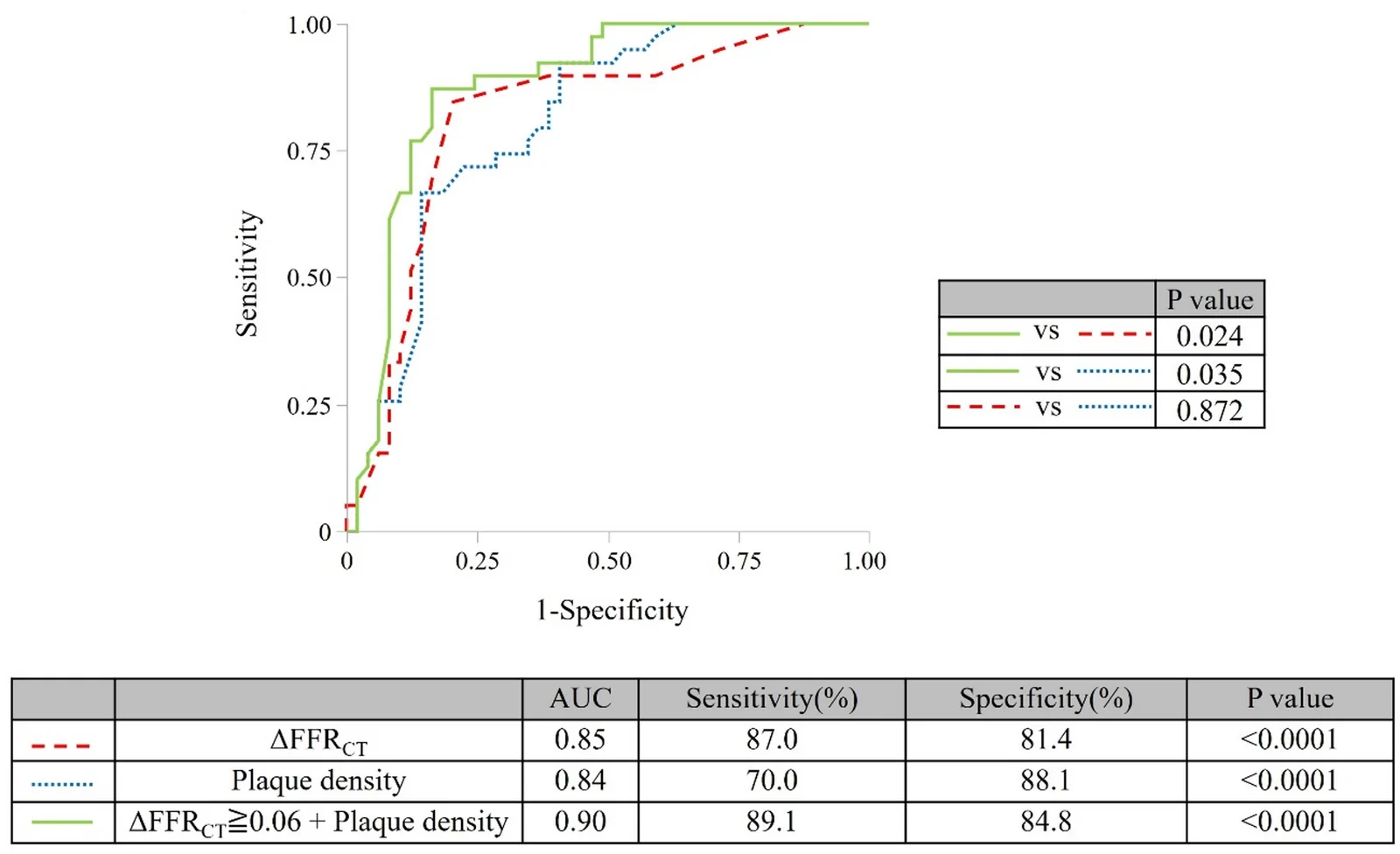
ROC curve analysis based on the combination of ΔFFR_CT_ ≥0.06 and plaque density for predicting a maxLCBI_4mm_ ≥400. A combination of ΔFFR_CT_ ≥ 0.06 and plaque density ≤ 29 HU on CCTA showed the highest diagnostic performance for noninvasive prediction of maxLCBI_4mm_ ≥ 400. AUC: area under the curve, FFR_CT_: fractional flow reserve derived from computed tomography

The model combining ΔFFR_CT_ ≥ 0.06 and plaque density ≤ 29 HU achieved the highest discriminative ability for identifying maxLCBI_4mm_ ≥ 400 (AUC = 0.90, sensitivity 89.1%, specificity 84.8%) (Fig. 5). There was no significant difference in AUC values between ΔFFR_CT_ and plaque density (0.85 vs. 0.84, *p* = 0.87). However, significant differences were observed between ΔFFR_CT_ and the combined model (0.90 vs. 0.85, *p* = 0.04) and between plaque density and the combined model (0.90 vs. 0.84, *p* = 0.02). The results of the univariate and multivariate analyses for predictors of maxLCBI_4mm_ ≥ 400 are presented in Table 3. Univariate analysis showed that plaque density ≤ 29 HU, percent diameter stenosis, positive remodeling, spotty calcification, napkin-ring sign, and ΔFFR_CT_ ≥ 0.06 were significantly associated with maxLCBI_4mm_ ≥ 400. In multivariate analysis, plaque density ≤ 29 HU, percent diameter stenosis, and ΔFFR_CT_ ≥ 0.06 were independent predictors of maxLCBI_4mm_ ≥ 400.

**Table 3:** Univariate and multivariate logistic analysis of predicting factors of maxLCBI_4mm_ ≧400

	Univariate analysis		Multivariate analysis	
	OR (95% CI)	*P* value	OR (95% CI)	*P* value
Plaque density ≦ 29	21.05 (7.53–58.81)	< 0.0001	7.72 (2.10-28.44)	0.002
Positive remodeling(RI ≧ 1.1)(+)	2.87 (1.16–7.11)	0.023	0.52 (0.11–2.36)	0.396
% Diameter stenosis (%)	1.12 (1.07–1.17)	< 0.0001	1.09 (1.01–1.16)	0.013
Spotty calcification(+)	3.91 (1.56–9.82)	0.004	2.07 (0.48–9.03)	0.332
Napkin –ring sign(+)	12.21 (1.47–101.60)	0.021	2.75 (0.13–59.43)	0.518
ΔFFR_CT_≧0.06	26.11 (8.98–75.88)	< 0.0001	4.55 (1.21–17.02)	0.025

## Discussion

To the best of our knowledge, this is the first study to evaluate the relationship between the addition of ΔFFR_CT_ values to CCTA-derived plaque features and maxLCBI_4mm_ in intermediate stenosis of non-culprit lesions in patients with ACS. The main finding of our study is that lesions with ΔFFR_CT_ ≥ 0.06 were significantly associated with maxLCBI_4mm_ ≥ 400, and that the addition of a plaque density ≤ 29 HU on CCTA further improved the diagnostic ability for identifying HRPs.

### Clinical implication of this study

Since the COURAGE trial, optimal medical therapy has been established as the standard treatment for chronic coronary syndrome (CCS) [14, 16, 17]. The results of this and the ISCHEMIA trial demonstrated that most patients with CCS can be managed conservatively with strict adherence to optimal medical therapy [18]. However, regarding the prognosis of patients with ACS, the GRACE registry reported mortality rates within 6 months of discharge of 4.8% for ST-segment elevation myocardial infarction, 6.2% for non-ST-segment elevation myocardial infarction, and 3.6% for unstable angina [19]. These rates remain higher than those for patients with CCS. Assessing the risk of non-culprit lesions in patients with ACS may therefore help reduce future cardiovascular events. The addition of FFR_CT_ has been shown to increase the positive predictive value for identifying functionally significant coronary lesions. The European Society of Cardiology and American Heart Association guidelines recommend CCTA as an initial imaging modality for patients with CCS [20, 21]. The present study suggests that FFR_CT_ is useful not only for assessing ischemia but also for predicting plaque vulnerability. In addition, recent studies have shown that intensive lipid-lowering therapy can favorably modify lipid-rich plaques, with lesions exhibiting maxLCBI_4mm_ ≥ 400 improving to < 400 over time, accompanied by improvements in coronary physiology as assessed by quantitative flow ratio [22]. In this context, although the present study was not designed to assess clinical outcomes or treatment effects, our findings suggest that non-invasive identification of lesions with high-risk plaque characteristics during the acute phase may help identify patients or lesions that could be considered for intensified lipid-lowering therapy and closer imaging follow-up. Furthermore, in cases where high-risk plaque features persist or progress despite optimal medical therapy, plaque-guided interventional strategies, as suggested by recent vulnerable plaque–focused trials such as the PREVENT trial, may be considered in selected patients, even in the absence of haemodynamically significant ischemia [11]. These concepts warrant further validation in prospective outcome-driven studies.

### Relationships between maxLCBI_4mm_ and HRPs

In the PROSPECT study, plaque burden > 70%, minimal lumen area < 4.0 mm², and the presence of thin-cap fibroatheroma were identified as independent risk factors for future events [23]. Plaques with these three characteristics had an event rate of approximately 18% over a 3-year period [23]. A comparative pathological study using NIRS-IVUS demonstrated that the sensitivity and specificity of NIRS-IVUS for detecting lipid-rich plaques were 90% and 93%, respectively [24]. In the PROSPECT II study, non-culprit lesions with maxLCBI_4mm_ ≥ 324.7 were associated with future cardiovascular events [10], and subsequent studies have consistently reported an association between maxLCBI_4mm_ ≥ 400 and HRPs [25]. In the present study, plaques with maxLCBI_4mm_ ≥ 400 identified by CCTA and NIRS-IVUS had greater plaque burden and area, and a smaller lumen area, compared with plaques with maxLCBI_4mm_ < 400.

### Relationships between maxLCBI_4mm_ and CCTA-derived plaque features

The CCTA features associated with HRPs have been reported as follows: (1) plaque density ≤ 30 HU, (2) positive remodeling (remodeling index ≥ 1.1), (3) presence of spotty calcification, and (4) presence of a napkin-ring sign [26]. A plaque density < 30 HU indicates a low-attenuation plaque, as shown in grayscale IVUS comparisons [27]. Several studies have attempted to define distinct HU ranges corresponding to histological plaque types [28]. A maxLCBI_4mm_ ≥ 400 is also associated with plaque density, and the optimal cutoff value for predicting maxLCBI_4mm_ ≥ 400 was previously reported to be 32.9 HU in coronary artery disease [29]. Our results are consistent with these findings, showing that a plaque density between 29 and 30 HU best predicted maxLCBI_4mm_ ≥ 400. Positive remodeling is another morphological feature of lipid-rich plaques [30]. In this study, positive remodeling was also associated with maxLCBI_4mm_. Moreover, lipid-rich plaques tend to show spotty calcification and a napkin-ring sign [27], which is consistent with our finding that plaques with maxLCBI_4mm_ ≥ 400 were more frequently calcified.

### Relationships between maxLCBI_4mm_ and FFR_CT_

Previous studies have reported that plaque vulnerability is associated with reduced FFR, although the underlying mechanisms are not yet fully understood [31]. Lipid-rich plaques develop through the accumulation of atherogenic inflammatory cytokines and oxidative stress, leading to endothelial dysfunction [32]. This concept is supported by evidence showing that lesions with large necrotic cores exhibit more severe endothelial dysfunction [31]. Because the endothelium regulates vascular tone [33], lipid atheromas with endothelial dysfunction may produce inadequate vasodilatory responses during hyperemia, leading to an increased ΔFFR.

FFR values are influenced by hydrodynamic factors such as wall shear stress, which is closely related to plaque morphology. As plaques become more unstable, wall shear stress increases and FFR decreases [28, 34]. Previous studies have shown that wall shear stress, axial plaque stress, and the pressure gradient correlate with ΔFFR_CT_, and there is a strong agreement between the sites of high shear stress and plaque rupture (κ = 0.79) [35]. Therefore, vulnerable plaques may influence ΔFFR_CT_ more strongly than stable plaques because of greater wall shear stress, axial plaque stress, and pressure gradients. Importantly, this conceptual framework is consistent with emerging pullback-based physiological indices, such as the pressure pullback gradient and derivative-based measures including dFFR/dt, which aim to characterize the spatial distribution and local intensity of pressure loss beyond conventional ischemia assessment. In this context, ΔFFR_CT_ may be interpreted as a non-invasive surrogate of focal hemodynamic disturbance related to plaque vulnerability rather than myocardial ischemia alone. In the present study, ΔFFR_CT_ ≥ 0.06 was an independent predictor of maxLCBI_4mm_ ≥ 400, and the combination with plaque density ≤ 29 HU further improved the diagnostic performance for detecting lipid-rich plaques. Thus, ΔFFR_CT_ may not only reflect the severity of local stenosis but also the morphological and biological characteristics of plaques. Notably, a similar magnitude of translesional ΔFFR_CT_ ≥ 0.06 has been reported in prior CCTA studies as a marker of adverse lesion-level hemodynamics preceding ACS, together with parameters such as elevated wall shear stress and axial plaque stress, supporting the physiological plausibility of our findings [26]. Given the relatively small sample size, the present study should be regarded as hypothesis-generating. Our findings provide exploratory evidence supporting the potential role of non-invasive hemodynamic and plaque-based imaging for identifying high-risk plaque phenotypes, which warrants confirmation in larger prospective studies.

In summary, ΔFFR_CT_ values correlated with maxLCBI_4mm_ and may enable non-invasive identification of HRPs. These findings may help develop noninvasive strategies to optimize lesion-specific pharmacological therapy and guide percutaneous coronary intervention.

### Study limitations

This study has several limitations.

First, it was a single-center study with a relatively small sample size; therefore, the findings should be interpreted with caution and considered hypothesis-generating.

Second, although 117 patients were initially enrolled, a substantial proportion were excluded because CCTA was not performed or FFR_CT_ analysis was not feasible, resulting in the inclusion of only 32 patients in the final analysis. Consequently, the analyzed cohort may represent a selected study population.

Third, the exact distance between the proximal and distal points used to calculate ΔFFR_CT_ was not fixed and may have varied depending on lesion length and vessel anatomy.

Forth, although the present study focused exclusively on non-culprit lesions, the potential utility of ΔFFR_CT_ in culprit lesions was not evaluated and requires dedicated investigation in future studies.

Fifth, because multiple lesions were analyzed within individual patients, intra-patient correlation cannot be completely excluded. Although lesion-based analyses are commonly used in intravascular imaging studies focusing on plaque characteristics, this methodological aspect may have influenced the statistical estimates [36–38].

Future multicenter studies with larger populations are warranted to confirm these findings.

## Conclusions

HRPs with a maxLCBI_4mm_ ≥ 400 can be accurately identified by confirming a plaque density ≤ 29 HU in lesions with ΔFFR_CT_ ≥ 0.06.

## Supplementary Information

Below is the link to the electronic supplementary material.

Supplementary Material 1
