# Supplementary material for: Non-invasive identification of high-risk plaques in non-culprit lesions of acute coronary syndrome
Source: Heart Vessels. 2026 Feb 16;41(9):590–601. doi: 10.1007/s00380-026-02663-6 (PMC13486015; doi:10.1007/s00380-026-02663-6)
Supplement: Supplementary file 1 [file 380_2026_2663_MOESM1_ESM.docx]

(1) **Supplemental Table 1**

|  | maxLCBI_4mm_ ≧400 (n=46) | maxLCBI_4mm_ <400 (n=59) | P　value |
| --- | --- | --- | --- |
| **Location of lesions** |  |  |  |
| **Target vessel** |  |  |  |
| LAD, n (%) | 20 (43.4%) | 25 (42.4%) | P=0.91 |
| LCX, n (%) | 13 (28.3%) | 14 (23.7%) | P=0.60 |
| RCA, n (%) | 13 (28.3%) | 20 (33.9%) | P=0.54 |
| **Target lesion location** |  |  |  |
| Proximal lesion, n (%) | 31 (67.4%) | 21 (35.5%) | P=0.001 |
| Mid lesion, n (%) | 11 (23.9%) | 27 (45.8%) | P-0.02 |
| Distal lesion, n (%) | 4 (8.7%) | 9 (15.3%) | P=0.31 |
| Far distal lesion, n (%) | 0 (0%) | 2 (3.4%) | P=0.21 |
| **CCTA findings at maxLCBI_4mm_ lumen site** |  |  |  |
| Vessel area(mm^2^, IQR) | 15.3 (11.3-18.9) | 12.0 (8.9-15.4) | P<0.01 |
| Lumen area(mm^2^, IQR) | 4.4 (2.9-6.9) | 5.1 (3.9-6.7) | P<0.01 |
| Plaque area(mm^2^, IQR) | 9.6 (7.1-13.4) | 7.0 (4.5-9.2) | P<0.001 |
| Plaque density, (HU, IQR) | 28 (21-41) | 55 (37-83) | P<0.0001 |
| % diameter stenosis, (%, IQR) | 50 (42-60) | 31 (25-45) | P<0.0001 |
| Remodeling index, (IQR) | 1.01 (0.95-1.14) | 1.00 (0.94-1.05) | P=0.05 |
| Spotty calcification, n (%) | 19 (41.3%) | 9 (15.3%) | P=0.003 |
| Napkin-ring sign, n (%) | 8 (17.4%) | 1 (1.7%) | P=0.004 |
| **NIRS-IVUS findings** |  |  |  |
| Plaque burden at the site of maxLCBI_4mm_ (%, IQR) | 69 (60-72) | 53 (44-63) | P<0.0001 |
| Minimal lumen area at the site of maxLCBI_4mm_ (mm^2^, IQR) | 4.5 (3.0-5.6) | 5.1 (3.8-7.4) | P=0.003 |
| Plaque area with minimal lumen area at the site of maxLCBI_4mm_ (mm^2^, IQR) | 9.6 (7.4-12.6) | 6.2 (4.2-9.5) | P<0.0001 |
| MaxLCBI_4mm_, (IQR) | 515 (431-628) | 253 (123-332) | P<0.0001 |
| **Functional indexes at the site of maxLCBI_4mm_** |  |  |  |
| ΔFFR_CT_ (IQR) | 0.1 (0.06-0.18) | 0.04 (0.02-0.05) | P<0.0001 |

Characteristics of CCTA, NIRS measures and Functional indexes between analyzed coronary lesions with and without maxLCBI_4mm_≧400

CCTA: Coronary computed tomography, CT: Computed tomography, FFR_CT_: Fractional flow reserve derived from computed tomography, IQR: Interquartile range, LAD: Left anterior descending, LCX: Left circumflex, maxLCBI4mm: Maximum 4-mm lipid-core burden index, NIRS-IVUS: Near-infrared spectroscopy and intravascular ultrasound, PCI: Percutaneous coronary intervention, RCA: Right coronary artery
